# Health shocks and changes in preventive behaviors: Results from the China Health and Retirement Longitudinal Study

**DOI:** 10.3389/fpubh.2022.954700

**Published:** 2022-07-27

**Authors:** Peng Zhang, Hongli Jiang, Wen Chen

**Affiliations:** School of Public Health, Fudan University, Shanghai, China

**Keywords:** health shocks, preventive behaviors, the China Health and Retirement Longitudinal Study, longitudinal studies, China

## Abstract

**Background:**

China is facing the challenge of rising prevalence and ballooning burden of chronic non-communicable diseases (NCDs); however, the Chinese middle- and older-aged population considerably lack preventive behaviors. Health shocks (HS), widely defined as sudden health deterioration brought on by diseases or accidents, bring a “teachable moment” to motivate changes in preventive behaviors.

**Objective:**

This study aims to examine the effect of HS on changes in preventive behaviors, including personal health practices and preventive care utilization.

**Methods:**

HS was defined as any five chronic disease diagnoses (cancer, heart disease, stroke, diabetes, and hypertension). The impacts of HS on smoking, drinking, and exercise, physical examination were estimated. The panel data of 13,705 respondents were obtained from the latest two waves of the China Health and Retirement Longitudinal Study (CHARLS) in 2015 and 2018. A multilevel propensity score match difference-in-difference (multilevel PSM-DID) model was constructed.

**Results:**

HS significantly decreased smoking (OR = 0.59, *p* < 0.05) and drinking (OR = 0.62, *p* < 0.01) and increased the utilization of auxiliary inspection in physical examination (OR = 1.19, *p* < 0.1). Major HS had significantly considerable and specific effects on reducing smoking and drinking (OR = 0.37 and 0.56, *p* < 0.01), while minor HS had relatively small effects on reducing smoking (OR = 0.74, *p* < 0.05) and drinking (OR = 0.69, *p* < 0.01), but extensive effects on initiating exercise (OR = 1.32, *p* < 0.05), physical examination (OR = 1.18, *p* < 0.1), and auxiliary inspection (OR = 1.30, *p* < 0.05).

**Conclusion:**

After HS, there is a teachable moment to promote positive changes in preventive behaviors. Guided by the 5A's brief intervention model (Ask, Advise, Assess, Assist, Arrange), tailored interventions should be targeted at these populations to reduce the risk of the progression and complications of existing diseases, prevent the related comorbidity, and prolong the expected life-span.

## Introduction

China is facing the challenge of rising prevalence and ballooning burden of chronic non-communicable diseases (NCDs) due to epidemiological transition and the fast growth of the elderly population with a much longer life expectancy (1). The top four leading causes of poor health and major disease burdens in China are cardiovascular disease, chronic obstructive pulmonary disease, cancer, and diabetes (2). It was estimated that in people aged over 40 years old, the rate of these diseases would double or even triple over the next two decades (3). The report from the World Bank also suggested that these diseases could be potentially prevented and managed with changes in unhealthy behaviors for aging people (4). These approaches to NCDs prevention and control include abstaining from tobacco smoking and alcohol drinking, promoting physical activity and early detection and treatment of diseases (5).

However, the Chinese middle- and older-aged population considerably lack preventive behaviors. In 2011, it was estimated that over 580 million Chinese have at least one modifiable behavior related to NCDs (3). Over 54% of Chinese men are current smokers, especially older people, which is among the highest in the world (3, 6). The high-average alcohol intake among Chinese people also indicates harmful alcohol abuse (4). Also, due to physical inactivity and fat intake, over 200 million people in China are overweight or obese, which ranks first globally (7). Additionally, preventive care services are still underutilized because people have low awareness of the importance of physical examinations, cancer screenings, etc., or have limited resources and accessibility.

China recognizes that it is a critical time to prevent and control NCDs and improve public health. Since the healthcare reform in 2009, China has been striving to launch major projects to promote basic public health services and manage NCDs (8). As one of the Basic Public Health Services, free basic physical examination is provided annually for Chinese elderly residents and delivered in community health centers to ensure they have access to general check-ups, basic surgical and internal medicine tests, and sense organ tests across the country. According to the health resources available in different areas, auxiliary inspection is available for free or with a small extra fee for residents. These exams include routine blood tests, urine tests, liver function tests, kidney function tests, lipids profile tests, blood glucose tests, electrocardiogram, chest fluoroscopy, etc. In 2016, the “Healthy China 2030 Scheme” was released with effective prevention and control strategies, such as extensively controlling smoking, reducing alcohol abuse, launching fitness programs, and promoting physical examinations and screenings (9). According to the 14th Five-Year Plan (2021–2025), China encourages personal health practices and prioritizes preventive care utilization (10).

Health shocks (HS) bring an important opportunity to motivate changes in preventive behaviors and promote strategy implementation. Health shock is widely defined as sudden health deterioration brought on by diseases or accidents (11). There are mainly six kinds of indicators to measure the occurrence of health shocks: newly diagnosed chronic disease diagnosis, the self-reported decline in health, limitation of daily activities, sudden increase in healthcare services utilization, catastrophic health expenditure, or abnormal change in body weight. Newly chronic disease diagnosis is more commonly recommended and used considering the exogeneity and suddenness of health shock in definition (12–15). Preventive behaviors are mostly defined as behaviors with the purpose of disease prevention and health promotion, for example, smoking cessation, alcohol use reduction, exercise, and active involvement in physical examination (16). In Andersen's health behavior model, health behaviors are divided into personal health practice, and health services use (17). Guided by this model, in this study, preventive behaviors were furtherly classified into two types: personal health practices, including tobacco and alcohol use, and exercise, and preventive care utilization, including utilization of physical examination, basic examination, and auxiliary inspection. Preventive care utilization is different from personal health practices because it requires the involvement of healthcare providers to provide preventive services, and is also affected by other factors, such as time constraints or accessibility (16). Health shock influences preventive behaviors as a wake-up call to warn people of the risk of potential diseases or complications and the importance of preventive behaviors. Then, health shock raises their attention to health and triggers changes in preventive behaviors (18, 19).

Recent research has indicated that health shock has a positive impact on changes in preventive behaviors in middle and older age. For personal health practices, global studies have proved that health shocks influenced smoking decisions and significantly decreased the probability of smoking and daily consumption of cigarettes (14, 18, 20–25). Only one research in China focused on the positive impact of health shocks on decreasing the likelihood of smoking (26). It is universally suggested that health shocks have a positive impact on controlling alcohol use. After health shocks of newly diagnosed chronic diseases, such as heart disease, diabetes, cancer, stroke, and lung disease, people changed their drinking habits with a significant decline in the probability of drinking and less alcohol consumption (24). Similarly, van Gool's study found significant associations between heart disease, as a health shock, with reduced alcohol use (23). The results of the effect of health shocks on engaging in exercise are mixed. Some studies indicated that after a new diagnosis of diabetes, people initiated more exercise, however other studies found people had no significant increase in participating exercise and oppositely even tended to be more sedentary after a new diagnosis of cancer or stroke. However, in China, personal health practices after HS are not well-studied. Local studies make no attempt to introduce multiple preventive behaviors at the same time to comprehensively estimate the overall effect of HS, so the changes in alcohol use and physical activity are unknown.

For preventive care utilization, little research recognized that compared to non-health-shock people, health-shock people utilize more preventive care services. Health shocks motivated people to participate in cholesterol tests and prompted women to take mammograms and men to take pap smears (27). Although following very serious health shocks, one-third of patients might temporarily suspend cancer screenings, the rates of screenings returned to normal within a year (28). Nevertheless, there is still a research gap between health shocks and changes in preventive care utilization in developing countries. Due to the limited accessibility of screening programs, physical examination is a more representative measure to reflect the utilization of preventive care services in China at this stage. As far as we know, no study analyzed the change of physical examination utilization with the further classification by its detailed items after health shocks.

This study aims to estimate the impact of health shocks on changes in preventive behaviors based on a nationally representative survey of the over 45-years-old population in China. HS was categorized into major HS and minor HS and further subdivided into multiple newly diagnosed diseases, including cancer, heart disease, stroke, diabetes, and hypertension to capture the heterogeneity. A multilevel propensity score match difference-in-difference (multilevel PSM-DID) model was constructed, and we examined the changes in a series of preventive behaviors, including personal health practices (smoking prevention, drinking reduction, and exercise) and preventive care utilization (physical examination, basic examination, and auxiliary inspection) after controlling related covariates. The main hypothesis is that health shocks trigger positive changes in preventive behaviors of the late middle- and older-aged population in China.

## Materials and methods

### Sample

The China Health and Retirement Longitudinal Study (CHARLS) is a nationally representative survey of the late middle- and older-aged population in China. Study samples were randomly selected from 28 provinces in mainland China through multistage probability-proportional-to-size sampling (29). The baseline survey was conducted in 2011, and 17,708 individuals from 10,026 communities were interviewed. The latest two follow-up waves were conducted in 2015 and 2018 and 21,095 and 19,816 individuals were followed up, respectively. Participants who dropped out because of migration or death were replaced with new respondents within the same family. Around 70% of the original 2011 sample were followed up through these waves, and the response rate was over 86%. CHARLS is designed and conducted complying with other related international studies (e.g., the Health and Retirement Study in America). The survey contains abundant information on demographic background, family information, health status, health care and insurance, employment, and economic conditions. The CHARLS is approved by the Ethical Review Committee of Peking University (29). Detailed information on the study design, sampling, survey content and process, data, and coding is available online (30).

The data in this study were obtained from the forward prior 2015 and 2018 waves of the CHARLS because more detailed items of physical examination were added after 2015, so we can distinguish between basic examination and auxiliary inspection (30). Data cleaning was conducted to ensure the raw data applicable to this study, including checking the logic of data and key measures, dealing with missing variables, and so on. The respondents who were 45 years old and above were chosen (*N* = 18,771). Seven hundred fifty-eight respondents were excluded because they only participated in the survey in the 2015 wave and their preventive behaviors could not be analyzed. Due to missing values of chronic disease diagnosis, 894 respondents were excluded. Finally, to reduce the confounding effects because of the prior diagnosis, we excluded chronic disease patients at baseline (*N* = 3,414). The final sample was comprised of 13,705 respondents.

### Measures

#### Health shocks

The key independent variables, health shocks, major HS, and minor HS, were whether there were any self-reported newly diagnosed chronic diseases between 2015 and 2018. The five most common chronic diseases selected were: cancer, stroke, heart disease, diabetes, and hypertension. Chronic diseases were assessed with the question “Have you been diagnosed with…” for the following list of conditions: “Cancer or malignant tumor (excluding minor skin cancers)?”; “Stroke?”; “Heart attack, coronary heart disease, angina, congestive heart failure, or other heart problems?”; “diabetes or high blood sugar?”; and “hypertension?”. Respondents who said “yes” in 2018 but previously said “no” in 2015 were identified as having experienced health shocks from 2015 to 2018. We examined health shocks for any one of five diseases, which are measured as a discrete indicator (1 if shocks occurred and 0 if not).

Furthermore, two categories of variables were divided to capture the heterogeneity. These classifications for major vs. minor shocks were adopted from literature (6, 27). Major HS included cancer, stroke, and heart disease, which are generally serious or even fatal diseases. Minor HS included diabetes and hypertension, which are normally long-term and not life-threatening. Then we coded major HS and minor HS for having any disease of three or two diseases listed above as discrete indicators (1 if shocks occurred and 0 if not). Lastly, we analyzed each disease of cancer, stroke, heart disease, diabetes, and hypertension to interpret the results. To identify the occurrence of health shock, if respondents were diagnosed with any six chronic diseases, they were included as the HS group. Individuals with multiple diseases were included in different models separately.

#### Preventive behaviors

The dependent variables were two sets of preventive behaviors: personal health practices and preventive care utilization, which were adopted from Andersen's health behavior model (17).

In this study, personal health practices reflected whether respondents performed health-related activities for a healthy lifestyle and better health status, such as smoking, drinking, and exercise. Three dependent variables were ascertained from questions asked for respondents at every wave: First, “Have you ever chewed tobacco, smoked a pipe, smoked self-rolled cigarettes, or smoked cigarettes/cigars?” and “Do you still smoke or have you totally quit?” were coded into a binomial variable of smoking defined by whether the non-smokers in 2015 started smoking in 2018 and whether the smokers in 2015 stopped smoking in 2018 (1 if smoking and 0 if not). Second, “Did you drink any alcoholic beverages, such as beer, wine, or liquor in the past year?” and a binomial variable of quitting drinking was conducted if the non-drinkers in 2015 started drinking in 2018 and the drinkers in 2015 stopped drinking in 2018 (1 if drinking and 0 if not). Lastly, questions of “Have you taken part in vigorous or moderate or mild activities at least 10 min every time in a week?” and “the purpose for doing these physical activities including for entertainment, job demand or exercise?” were used and a binomial variable of exercise generated as respondents took part in any kind of activities only for the purpose of the exercise (1 if exercise and 0 if not).

Preventive care utilization implied whether respondents used preventive care services. In our study, physical examination was further classified into two kinds: basic examination and auxiliary inspection. At every wave, participants were asked: “whether they take any physical examination not for the treatment purpose and which items do they take in this physical examination.” According to the National Code of Basic Public Health Services in China, the basic examination was defined as free tests not involving medical devices, including general check-ups, surgical tests, internal medicine tests, and sense organ tests. Auxiliary inspection was defined as laboratory tests requiring the support of medical devices, including routine blood tests, routine urine tests, liver function tests, kidney function tests, lipids profile tests, blood glucose tests, electrocardiogram, chest fluoroscopy, etc. We coded these into three binomial variables defined by whether they utilized any physical examination, basic examination, or auxiliary inspection within a year before the interview in 2015 and 2018 (1 if used and 0 if not).

#### Covariates

According to contextual and individual characteristics in Andersen's health behavior model, we included the four sets of covariates associated with preventive behaviors in our model (17). First, individual predisposing variables included age, gender, minority, marital status, education level, occupation, and urban/rural location. Second, enabling variables consisted of health insurance type and annual household expenditure level. Household expenditure level was more accurate than self-reported income in this survey and was coded into three quartiles due to skewness. Third, the need variable was represented by self-reported health status, which was measured by the question, “Would you say your health is very good, good, fair, poor or very poor?”. The results were coded into three levels by keeping “fair” as the middle level, and combining “very good” and “good” into the high level, and “very poor” and “poor” into the low level. Lastly, contextual variables were controlled by regional location (East, Mid, and West areas of China) to reflect some unobserved factors. All these covariates in 2015 were used as matching factors at baseline and covariates in 2015 and 2018 were controlled in multivariate models.

### Statistical analysis

A descriptive analysis was employed to report preventive behaviors in 2015 and 2018 and covariates at baseline. To estimate the effects of health shocks on preventive behaviors, a multilevel PSM-DID model was constructed (31, 32). Before constructing the multilevel DID model, we inspected the assumption of “parallel trends.” However, due to the limited data with the short time span of two waves available of CHARLS in our study, the preliminary analysis cannot be conducted because of collinearity. To better solve this issue, the PSM method was applied to balance the two groups at baseline, as well as possible to make this assumption plausible (33). After PSM, because of the similarity of other characteristics, it was assumed that determinants of outcomes remained stable in the two groups over time. The matching variables used in PSM were age, gender, ethnicity, marital status, education level, occupation, household expenditure, self-reported health status, and regional location, which were suggested by the Andersen's health behavior model and associated with preventive behaviors. After comparing the matching results of four different methods by the reduced bias and *t*-test of each variable before and after matching (see Appendices A–D), kernel matching with logit regression was selected as the best method with a bandwidth of 0.06. In the sensitivity analysis, we used the 1:3 nearest matching, the second-best method, as the PSM method combined with the multilevel DID model to estimate the effects.

Then, the DID model was adopted using the following model in Equation (1) and estimated using the logit method (19, 34). The basic DID model is a rigorous approach, as a quasi-experimental design, to simulate the counterfactual scenario of how preventive behaviors would have been if HS had not occurred and compared the changes in preventive behaviors between the HS group and the non-HS group to estimate the effect of HS. Indicated by the dummy variable “*HS*_*ijk*_,” individual *i* was classified as the treatment group or the control group. For example, the treatment group of HS implied health-shocks individuals who had any five diseases for “1” and the control group of HS included the non-health-shocks individuals who were free of any diseases for “0.” The year 2015 was defined as the pre-treatment period and 2018 as the post-treatment period when the dummy variable “*time*_*ijk*_” equals to “1” and “0”, respectively. For six outcomes (*Y*_*ijk*_), the average treatment effect was estimated by comparing the probability change of the treatment group with the probability change of the control group from 2015 to 2018. The parameter of interest was the coefficient of the interaction term, β_1_, which would capture the different probability changes in *Y*_*ijk*_ among patients in two groups if health shocks had an impact. *X*_*ijk*_ consisted of covariates listed in Table 1.

**Table 1:** Individual characteristics of the study sample from CHARLS after kernel matching.

	**Total**		**Health-shocks**		**Non-health-shocks**	
	**N**	**%**	**N**	**%**	**N**	**%**
	13,705	100.00	9,004	100.00	4,701	100.00
*Individual predisposing variables*				
Age (yrs)						
45–59	6,550	47.79	3,646	40.49	2,904	61.77
60–74	5,932	43.28	4,408	48.96	1,524	32.42
75–89	1,206	8.80	943	10.47	263	5.59
90+	17	0.12	7	0.08	10	0.21
Gender						
Male	6,670	48.67	4,238	47.07	2,432	51.73
Female	7,035	51.33	4,766	52.93	2,269	48.27
Han population						
Yes	12,751	93.04	4,438	49.29	8,313	176.83
No	954	6.96	263	2.92	691	14.70
Married						
No	1,635	11.93	1,180	13.11	455	9.68
Yes	12,070	88.07	7,824	86.89	4,246	90.32
Education level					
No formal education	5,749	41.95	4,023	44.68	1,726	36.72
Elementary school	3,047	22.23	2,010	22.32	1,037	22.06
Secondary school	3,179	23.20	1,903	21.14	1,276	27.14
High school	1,488	10.86	912	10.13	576	12.25
College and above	242	1.77	156	1.73	86	1.83
Occupation					
Others	1,130	8.25	740	8.22	390	8.30
Farmer	10,206	74.47	6,578	73.06	3,628	77.18
Civil servant	487	3.55	290	3.22	197	4.19
Retired	1,882	13.73	1,396	15.50	486	10.34
Urban/rural location					
Rural	10,874	79.34	7,011	77.87	3,863	82.17
Urban	2,831	20.66	1,993	22.13	838	17.83
*Individual enabling variables*				
Health insurance type					
No insurance	312	2.28	198	2.20	114	2.43
UEBMI	11,334	82.70	7,368	81.83	3,966	84.37
URRBMI	2,059	15.02	1,438	15.97	621	13.21
Household expenditure				
Lowest quantile	4,027	29.38	2,690	29.88	1,337	28.44
Middle quantile	4,853	35.41	3,176	35.27	1,677	35.67
Highest quantile	4,825	35.21	3,138	34.85	1,687	35.89
*Individual need variable*					
Self-reported health status				
Lowest level	5,107	37.26	4,282	47.56	825	17.55
Middle level	5,279	38.52	3,428	38.07	1,851	39.37
Highest level	3,319	24.22	1,294	14.37	2,025	43.08
*Contextual variables*					
Regional location					
East area	4,911	35.83	2,949	32.75	1,962	41.74
Mid area	4,616	33.68	3,164	35.14	1,452	30.89
West area	4,178	30.49	2,891	32.11	1,287	27.38

*Urban and rural resident medical insurance (UEBMI) integrates previous urban resident medical insurance and new rural cooperative medical insurance for informal employees and residents in China. UEBMI, urban and rural resident medical insurance. URRBMI, urban employee medical insurance*.

The national survey of the cohort with multistage sampling contains information about the respondents, who were repeatedly observed in different periods and simultaneously clustered at different levels. As a result, the dependence on data often, appears and the outcomes of individuals within the same level could have more similarities than those from the different levels. To capture the unmeasured variations within every level and correctly estimate the standard errors, a multilevel model was widely constructed and combined with the basic DID model (35, 36). We conducted multilevel random intercept analyses with the three-level structure of individual-wave-level, individual-level, and community-level, because similar studies based on multistage sampling stressed the random effects attributed to context effects of different waves and communities (35, 36). Subscripts *i*, *j*, and *k* corresponded, respectively, to individual-wave-level, individual-level, and community-level of the model. This multilevel DID model estimates not only the fixed effects reflecting the relationship between observed variables and the outcomes, but also the random effects representing effects from variations among waves, individuals, and communities (31, 35).


(1) $$\begin{array}{l} ln(\frac{PY_{ijk}}{1-PY_{ijk}})=\alpha_{ijk}+\beta_{1}{\left(HS^{*}time\right)}_{ijk}+\beta_{2}{\left(HS\right)}_{ijk}\;\\ \;+\beta_{3}{\left(time\right)}_{ijk}+\beta_{4}{\left(X\right)}_{ijk}\; \end{array}$$

(2) $$\begin{array}{r} \alpha_{ijk}=\alpha +v_{k}+u_{jk}+e_{ijk} \end{array}$$

(3) $$\begin{array}{r} v_{k}\sim N\left(0,\sigma_{v}^{2}\right),u_{jk}\sim N\left(0,\sigma_{u}^{2}\right),e_{ijk}\sim N\left(0,\sigma_{e}^{2}\right) \end{array}$$

(4) $$\begin{array}{r} cov\left(v_{k},u_{jk},e_{ijk}\right)=0 \end{array}$$

(5) $$\begin{array}{r} var\left(Y_{ijk}\right)=\sigma_{v}^{2}+\sigma_{u}^{2}+\sigma_{e}^{2} \end{array}$$

The multilevel PSM-DID models were estimated in Stata 15.0 SE (StataCorp LLC, College Station, TX, USA) using melogit command with robust standard errors, as well as descriptive analysis. The threshold of statistical significance was at *P* < 0.05 (two-tailed).

## Results

### Sample characteristics

The individual characteristics of the study sample from CHARLS after kernel matching are presented in Table 1. Before matching, the difference between these characteristics at baseline was statistically significant, whereas after kernel matching with logit regression, the two groups were statistically well-balanced except for two variables (see Appendix A). A total of 13,750 respondents were included. At baseline, the average age of our sample was 60.78, with a majority of females (51.33%), the Han populations (93.04%), and married people (88.07%). A total of 41.95% of respondents lacked formal education, most of them worked in agriculture (74.47%) and lived in rural areas (79.34%). Only 2.28% were uninsured, the other 82.70% of respondents had urban and rural resident medical insurance (UEBMI) for informal employees and residents, and 15.02% of respondents had urban employee medical insurance (URRBMI) for formal employees, including retirees. Self-reported household expenditure was divided into three quartiles: lowest, middle, and highest. A total of 37.26% of participants self-reported having poor or very poor health as the lowest level, while 24.22% self-reported having good or very good health as the highest level. The regional location of our sample was evenly distributed, with a slightly higher proportion in the East area (35.83%).

There were 9,004 respondents in the health-shocks group and 4,701 respondents in the non-health-shocks group. As compared to the non-health-shocks respondents, the health-shocks respondents were more likely to be elderly people with worse health status. More of the health-shocks respondents were married females and minority populations. They also tended to be without formal education and already retired. Additionally, more health-shocks respondents had insurance with a lower level of household expenditure and lived in Mid or West urban areas.

### Health shocks and preventive behaviors

The occurrence of health shocks during follow-up in this study sample is presented in Table 2. From 2015 to 2018, health shocks occurred to 9,004 (65.70%) among 13,705 respondents. A total of 27.61% of our sample suffered from major HS and 35.05% experienced minor HS. To be specific, 25.88% of the respondents reported hypertension diagnosis, followed by heart disease (16.73%), diabetes (12.11%), stroke (10.15%), and cancer (2.71%).

**Table 2:** The occurrence of HS during 2015 to 2018 and preventive behaviors before and after HS in different groups.

	**The occurrence** **of HS**		**Preventive behaviors**											
			**Smoking**		**Drinking**		**Exercise**		**Physical examination**		**Basic examination**		**Auxiliary inspection**	
**Groups**	**N**	**%**	**2015**	**2018**	**2015**	**2018**	**2015**	**2018**	**2015**	**2018**	**2015**	**2018**	**2015**	**2018**
Total	13,70	100.0	28.6	27.0	27.2	26.4	26.8	36.0	40.6	47.5	27.8	37.7	36.5	45.8
Non-HS	4,701	34.30	32.8	32.40	30.5	32.0	21.8	28.6	30.7	35.7	20.7	28.2	27.4	33.7
HS	9,004	65.70	26.5	24.2	25.5	23.5	29.3	39.8	45.8	53.8	31.5	42.8	41.3	52.1
Major HS	3,784	27.61	24.3	20.6	22.5	19.5	31.0	41.5	47.4	55.1	32.5	43.0	43.2	53.3
Cancer	372	2.71	26.6	12.7	21.5	15.7	32.5	39.5	48.7	56.3	34.2	45.4	46.1	55.1
Stroke	2,293	16.73	24.6	21.6	21.9	17.4	35.4	47.4	50.2	58.4	35.8	45.0	44.1	56.6
Heart disease	1,391	10.15	23.7	21.2	23.1	20.5	28.4	38.5	46.9	54.1	31.0	42.6	43.1	52.1
Minor HS	4,803	35.05	28.2	25.9	27.6	25.3	26.3	38.5	43.8	52.7	30.0	42.4	39.1	51.2
Diabetes	1,660	12.11	24.5	22.7	24.8	21.7	30.4	43.5	48.2	58.4	31.3	46.3	44.1	57.3
Hypertension	3,547	25.88	29.5	27.0	28.8	26.7	24.6	37.3	41.7	51.0	29.3	41.7	36.8	49.4

*HS, health shocks; Non-HS, non health shocks*.

Table 2 also shows the prevalence of engagement in preventive behaviors before and after health shocks in the health-shocks and the non-health-shocks groups. Generally, from 2015 to 2018, the over 45-years-old population in China slightly decreased smoking and drinking and increased exercise. Also, they participated in more physical examinations, both basic examinations and auxiliary inspections. In the different classifications of health shocks, the patterns of change in preventive behaviors after HS were similar. For participants with health shocks, the rates of smoking declined after health shocks, with a range of 1.76% in diabetes to 13.84% in cancer. Similarly, the rates of drinking in the HS group also declined after health shocks, with a range of 2.07% in any HS to 5.80% in cancer. The rates of exercise in HS participants and the rates of utilization of physical examination, basic examination, and auxiliary inspection increased in 2018, with the biggest rise of 13.11, 10.18, 15.02, and 13.18% in diabetes. For participants without health shocks, the personal practices were worse than participants with HS. The rates of smoking remained almost the same in 2015 as in 2018, and the rates of drinking were 1.15% higher in 2018. 6.85% more non-HS participants engaged in an exercise in 2018 than in 2015, and 4.98, 7.45, and 6.32% more non-HS participants used preventive care. The rises in the percentage of exercise and utilizing preventive care in the non-health-shocks group were smaller than in the health-shocks group.

### Multilevel PSM-DID regression

The results of multilevel PSM-DID regressions are shown in Table 3. After propensity matching using the kernel matching method with logit regression, the characteristics of the two groups were quietly balanced at baseline (see Appendix A). Six multilevel DID regressions were conducted, and the net effect of HS on changes in preventive behaviors was presented by the interaction terms of the first row corresponding to different shocks. From logistic regression after adjusting for individual characteristics, the odds ratios were displayed to reflect the odds of involving in a particular preventive behavior. An odds ratio >1 indicates an increased likelihood of preventive behaviors, whereas an odds ratio <1 suggests a decreased likelihood of preventive behaviors after HS. The stars show different levels of statistical significance.

**Table 3:** Odds ratios from multilevel PSM-DID regression models predicting the effect of health shocks on preventive behaviors.

**Health shocks**	**Smoking**	**Drinking**	**Exercise**	**Physical examination**	**Basic examination**	**Auxiliary inspection**
HS × Time	0.59^**^	0.62^***^	1.18	1.12	1.11	1.19^*^
HS	0.06^***^	0.84^**^	1.43^***^	2.01^***^	1.82^***^	2.03^***^
Time	0.80	1.11	1.50^***^	1.28^***^	1.60^***^	1.44^***^
Major HS × Time	0.37^***^	0.56^***^	1.20	1.11	1.07	1.14
Major HS	0.10^***^	0.64^***^	1.47^***^	2.31^***^	2.02^***^	2.34^***^
Time	0.84	1.11	1.50^***^	1.25^***^	1.59^***^	1.41^***^
Minor HS × Time	0.74^**^	0.69^***^	1.32^**^	1.18^*^	1.21	1.30^**^
Minor HS	0.43	0.91	1.29^*^	1.86^***^	1.69^***^	1.85^***^
Time	0.86	1.17	1.48^***^	1.27^***^	1.62^***^	1.43^***^
Cancer × Time	0.03^**^	0.41	0.82	1.08	1.13	1.06
Cancer	0.02^***^	0.44^*^	1.76	2.29^***^	2.12^***^	2.59^***^
Time	0.89	1.14	1.51^***^	1.22^***^	1.61^***^	1.392^***^
Stroke × Time	0.54^***^	0.41^***^	1.28	1.16	0.97	1.32^*^
Stroke	0.05^***^	0.38^***^	1.90^***^	2.82^***^	2.45^***^	2.54^***^
Time	0.93	1.09	1.47^***^	1.22^***^	1.58^***^	1.39^***^
Heart disease × Time	0.60^**^	0.56^***^	1.26	1.10	1.16	1.09^*^
Heart disease	0.35^***^	0.81	1.24	2.31^***^	1.99^***^	2.43^***^
Time	0.86	1.15	1.46^***^	1.25^**^	1.60^***^	1.42^***^
Diabetes × Time	0.67	0.72^***^	1.32^**^	1.28^*^	1.39^*^	1.37^**^
Diabetes	0.14^***^	0.69	1.59^**^	2.48^***^	1.84^***^	2.58^***^
Time	0.94	1.17	1.47^***^	1.22^***^	1.58^***^	1.39^***^
Hypertension × Time	0.63^**^	0.59^***^	1.40^***^	1.24^*^	1.25^*^	1.37^**^
Hypertension	0.18^***^	0.99	1.17	1.68^***^	1.66^***^	1.63^***^
Time	0.87	1.17	1.49^***^	1.27^***^	1.64^***^	1.44^***^

*The multilevel PSM-DID model was conducted by adjusting age, gender, ethnicity, marital status, education level, occupation, household expenditure, self-reported health status, and regional location, and estimated at individual-wave-level, individual-level, and community-level. HS, health shocks*.

* *p < 0.05*.

** *p < 0.01*.

*** *p < 0.001*.

*The bold values show the parameters of interest in every multilevel PSM-DID model, which is the coefficient of the interaction term β_3_ in every first line*.

Compared to non-HS participants, participants with HS ceased harmful behaviors and prompted exercise and utilized more preventive care. After health shocks, people significantly decreased smoking, and the odds ratio was 0.59 times less in HS people than in non-HS people. Similarly, people in the HS group were more likely to cease drinking habits, and the difference was significant (OR = 0.62). More people exercised regularly and took the physical examinations, and the odds ratio was significant in the increase of auxiliary inspection (OR = 1.19).

The difference appeared in the change of preventive behaviors after major health shocks and minor health shocks. Both major HS and minor HS significantly decreased smoking and drinking, but major HS had a bigger effect on behavior changes. The odds ratios of smoking and drinking were 0.37 and 0.56 after major HS, and 0.74 and 0.69 after minor HS. Nevertheless, after minor HS the probability of exercise significantly increased compared with the non-health-shocks group (OR = 1.30), but no significant increase in exercise was observed after major HS. After major HS or minor HS, more physical examinations, including basic examinations and auxiliary inspections, were used than in the non-shocks group; however, only after minor HS the increases in a physical examination and auxiliary inspection utilization were significant (OR = 1.18 and 1.30).

To be specific, we analyzed preventive behaviors change after different diagnosed disease diagnosis. Cancer significantly and dramatically decreased smoking (OR = 0.03) but had no influence on other preventive behaviors. Participants diagnosed with stroke and heart disease had a significantly lower probability of smoking (OR = 0.54 and 0.60) and drinking (OR = 0.41 and 0.56). After a stroke diagnosis, they also utilized more auxiliary inspection (OR = 1.32). A diabetes diagnosis was associated with less drinking (OR = 0.72), more exercise (OR = 1.32), and more utilization of physical examination, basic examination, and auxiliary inspection (OR = 1.28, 1.39, and 1.37), which were all statistically significant. No significant decline in smoking was associated with the occurrence of diabetes. The changes in preventive behaviors were most extensive after a hypertension diagnosis. After a hypertension diagnosis, patients not only reduced smoking and drinking (OR = 0.63 and 0.59), but also increased physical examination, basic examination, and auxiliary inspection (OR = 1.24, 1.25, and 1.37). Significantly, HS participants began regular physical activity after being diagnosed with hypertension (OR = 1.40).

For the sensitivity analysis (see Appendix E), after the 1:3 nearest matching, the odds ratios from multilevel PSM-DID regression models predicting the effect of health shocks on preventive behaviors indicated slightly bigger and still significant estimates for all the outcome measures, so the main results withstood the sensitivity test and showed robust results.

## Discussion

This is a plausible study to prove that in China, HS initiates positive changes in multiple preventive behaviors in the late middle- and older-aged population with outcome measures of personal health practices and preventive care utilization. It adds to global literature that HS promotes preventive care utilization like basic physical examination and reveals the heterogeneity in the impact of major HS and minor HS.

In summary, health shocks from any of the five chronic disease diagnoses (cancer, heart disease, stroke, diabetes, and hypertension) significantly decreased the probability of smoking and drinking, and alsoincreased the probability of involving in the auxiliary inspection. The high occurrence of HS in the Chinese late middle- and older-aged population stressed the importance to consider HS as a wake-up call to study its effect on positive preventive behaviors. The role of HS in preventive behavior changes could be explained by the following reasons. For internal reasons, after health shocks, people perceived more risk of disease and noticed the importance of preventive behaviors to maintain health (37). Health shocks occurred as “a trigger” to motivate people to change their lifestyles and utilize preventive services, which were more targeted and effective than learning from health shocks on others or related educational materials (18). For external reasons, healthcare providers and social networks play significant roles to ensure behavior changes. There is a “teachable moment” after a new disease diagnosis when patients are more concerned about their health and receptive to health information. At the same time, the frequency of communicating with health care providers increased for people with health shocks. It is a valuable opportunity for providers to advise and assist patients to optimize their preventive behaviors (24, 38, 39). Also, social networks like family members, friends, or even colleagues support and supervise people with health-shocks to start preventive behaviors (18, 23, 27, 37).

Health shocks have a positive effect on changes in personal health practices. In consistence with former studies, HS significantly decreased the probability of smoking and drinking (14, 22–24, 40). In our study, all specific disease diagnoses reduced smoking habits except for diabetes diagnosis. It suggested that people were more familiar with the risk of smoking and paid more attention to their smoking habits, because the correlation between smoking and health damage is more solid and well-known (24, 40). As for drinking habits, our study also suggested that health shocks reduced the probability of drinking in China as former global studies (23, 24). In contrast to earlier findings, however, no evidence of a reduction in drinking after cancer diagnosis was detected (24). There is a reasonable explanation that due to the discomfort and stress related to diseases, such as cancer in our case, patients may distract themselves by maintaining some addictive habits (38). We found that a new diagnosis of hypertension, besides diabetes in a former study, initiated significantly more involvement in exercise (41). These two diseases may be more closely related to sedentary lifestyles, and complications could be prevented by exercise.

It is also newly discovered that after HS, people utilized more preventive care services, especially auxiliary inspection. Scarce evidence suggests that HS initiates people to use preventive services, and this study mainly focused on the positive change in cancer screenings in the US (27). However, in developing countries like China, the more common and accessible preventive service is physical examination rather than cancer screenings. Our results first indicated a positive change of involvement in more physical examinations after HS. In general, people with HS significantly used more auxiliary inspection with laboratory tests, rather than a basic examination with free tests like general check-ups. The late middle- and old-aged adults with newly diagnosed diseases interacted more with healthcare providers and were educated with more professional health information during HS. At the same time, healthcare providers may suggest HS people to take specific tests for follow-up care for the purpose of managing diseases. As a result, they updated their knowledge of the necessity of taking related preventive services and were effectively spurred to use services recommended by providers (27).

There was heterogeneity in the impact of major health shocks and minor health shocks. Major health shocks (cancer, heart disease, and stroke) only decreased the probability of smoking and drinking with significantly considerable effect, while did not influence the change of exercise or preventive care utilization. In consistence with other studies, major HS seriously hit people with the urgency to change behaviors (23, 24, 39). For example, a new diagnosis of cancer, stroke, and heart disease had a much bigger effect than hypertension or diabetes on decreasing smoking. In other words, the effect on preventive behaviors may be positively correlated with the severity of the disease so that people who suffer from major diseases, tend to perceive more risks of harmful lifestyles and a stronger connection between disease diagnosis and health practices. However, after major HS, people tend to perceive limited benefits so they make less effort to prolong their life by comprehensively changing multiple behaviors. This circumstance might be interpreted by people's life expectancy, which was heavily deteriorated by serious health events (28). Additionally, people with major diseases like cancer and stroke mainly receive rigorous treatment rather than preventive care, so they have restricted time to prevent other diseases.

Although minor health shocks were mainly long term and not life-threatening chronic diseases (diabetes and hypertension), it had a more extensive impact on multiple preventive behaviors. Minor HS had significant but relatively small effects on reducing smoking and drinking; it also significantly triggered participating in exercise, physical examination, and auxiliary inspection. Analyzing each disease, we found that hypertension triggered the widest change of all six preventive behaviors. After hypertension diagnosis, patients not only decreased smoking and drinking and increased exercise, but also participated in more physical examinations and utilized more basic examinations and auxiliary inspections. This indicates people with minor HS have a higher level of self-efficacy to prevent complications and other diseases by adopting better lifestyles (42).

This study has some strengths. First, based on the nationally representative survey with the panel data of 13,705 respondents, this study newly proved that in China, health shocks aroused over 45-years-old population to improve their preventive behaviors. Second, our study also demonstrated the heterogeneity in the impacts of major health shocks and minor health shocks by classifying and analyzing different diseases. Last but not the least, besides the usual outcome measures of personal health practices, we introduced physical examination and its two kinds as more suitable measures to represent the most widespread preventive services in developing countries. The results make a novel contribution to proving the significant increase in the utilization of physical examination, especially auxiliary inspection after health shocks.

The limitations of this study should be mentioned. Only follow-up data after the 2015 waves of CHARLS added the detailed physical examination items, so the observation period is limited, and the preliminary analysis cannot be conducted. We applied the PSM method to balance two groups to solve this problem and ensure the balance between two groups. Then, we could only observe the change of behaviors after health shock in the short term, while the maintenance of these behaviors should be observed in the future. Additionally, the self-reported preventive behaviors might have recall bias, and participants tend to overreport their positive preventive behaviors.

These results also have some policy implications. Our results proved there is a teachable moment after health shocks when patients are informed by health risks and are more receptive to health information. They are not only threatened by the complications and recurrences of health shock caused by the present diseases, but also susceptible to other chronic diseases in the future. To reduce the risk of the progression and complications of existing diseases, prevent the related comorbidity, and prolong the expected life-span, health shocks provide healthcare providers a great chance to better adopt the 5A's brief intervention model (Ask, Advise, Assess, Assist, and Arrange) as guidelines for initiating preventive behaviors (43, 44). Specifically, when health shocks occur, providers should ask patients about their preventive behaviors and provide more targeted health information. Then, shortly after newly diagnosed diseases, providers should seize this opportunity to propose health advice to trigger preventive behavior changes. At the same time, they should make an assessment of patients' motivation and barriers. Furthermore, based on the specific chronic diseases and assessment results, more tailored assistance and customized arrangements should be promoted for newly health-shock patients.

## Conclusion

To evaluate the impact of health shocks on changes in preventive behaviors among the over 45-years-old population in China, this article focused on a multilevel propensity score match difference-in-difference model based on a nationally representative survey, CHARLS. It is proved that health shocks trigger positive changes in preventive behaviors by significantly decreasing the probability of smoking and drinking and increasing the probability of taking auxiliary inspection in physical examination. After health shocks, interventions should be targeted at these populations during the teachable moment to promote preventive behaviors and improve health.

## Data availability statement

Publicly available datasets were analyzed in this study. This data can be found at: https://charls.pku.edu.cn/en/.

## Ethics statement

Ethical approval for all the CHARLS waves was granted from the Institutional Review Board at Peking University. The IRB approval number for the main household survey including anthropometrics was IRB00001052-11015 and the IRB approval number for biomarker collection was IRB00001052-11014. The patients/participants provided their written informed consent to participate in this study.

## Author contributions

PZ contributed to the conception and design of the study under the supervision of WC. PZ performed the data collection, conducted the statistical analysis, wrote the manuscript, and revised it as needed under the guidance of WC and HJ. WC supervised the overall study and provided feedback throughout all stages. All authors approved the submitted version.

## Conflict of interest

The authors declare that the research was conducted in the absence of any commercial or financial relationships that could be construed as a potential conflict of interest.

## Publisher's note

All claims expressed in this article are solely those of the authors and do not necessarily represent those of their affiliated organizations, or those of the publisher, the editors and the reviewers. Any product that may be evaluated in this article, or claim that may be made by its manufacturer, is not guaranteed or endorsed by the publisher.
