# Supplementary material for: Health shocks and changes in preventive behaviors: Results from the China Health and Retirement Longitudinal Study
Source: Front Public Health. 2022 Jul 27;10:954700. doi: 10.3389/fpubh.2022.954700 (PMC9363769; doi:10.3389/fpubh.2022.954700)
Supplement: Supplementary file 1 [file Data_Sheet_1.pdf]

Supplementary Material

Health Shocks and Changes in Preventive Behaviors：Results from the China Health and Retirement Longitudinal Study

Appendix A Individual Characteristics of the Study Sample from CHARLS before and after Kernel Matching

		Mean				t-test	
**Variable**		**HS**	**non-HS**	**% Bias**	**%reduct |bias|**	**t**	**p>|t|**
**Age**	Before	60.84	56.35	48.50		19.20	0.00
	After	60.84	60.81	0.30	99.40	0.14	0.89
**Gender**	Before	0.53	0.48	9.20		3.67	0.00
	After	0.53	0.51	4.60	49.60	2.23	0.03
**Han population**	Before	0.08	0.06	8.50		3.33	0.00
	After	0.08	0.08	0.50	94.00	0.23	0.82
**Married**	Before	0.88	0.91	-11.40		-4.44	0.00
	After	0.88	0.87	2.30	80.10	1.01	0.31
**Education level**	Before	1.01	1.20	-16.60		-6.63	0.00
	After	1.01	1.00	1.30	91.90	0.65	0.51
**Occupation**	Before	1.38	1.24	13.70		5.34	0.00
	After	1.38	1.36	2.00	85.10	0.93	0.35
**Urban/rural location**	Before	0.22	0.18	11.10		4.35	0.00
	After	0.22	0.20	5.20	53.10	2.44	0.02
**Health insurance type**	Before	1.13	1.10	8.10		3.21	0.00
	After	1.13	1.12	3.00	62.50	1.44	0.15
**Household expenditure**	Before	2.04	2.07	-3.40		-1.36	0.17
	After	2.04	2.03	2.10	39.90	0.99	0.33
**Self-reported health status**	Before	0.57	1.09	-68.00		-27.67	0.00
	After	0.57	0.58	-1.00	98.50	-0.53	0.60
**Regional location**	Before	0.99	0.85	16.70		6.69	0.00
	After	0.99	0.98	1.30	92.40	0.61	0.54

The order “pstest” in STATA was used to compare individual characteristics between health-shocks respondents and the non-health-shocks respondents before and after propensity score matching.
The first line of each variable showed before matching the distribution, the bias and t-test results of the difference between two groups, which indicated that all the variables except household expenditure were statistically different.
The second line of each variable showed after matching the distribution, the bias and t-test results of the difference between two groups. After matching, all the bias of 11 matching variables were less than 10 (one of the standards of well-balanced) and the t-test results indicated that all the variables except 2 variables (gender and urban/rural location) were not statistically different.

Appendix B Individual Characteristics of the Study Sample from CHARLS before and after 1:1 Nearest Matching

		Mean				t-test	
**Variable**		**HS**	**non-HS**	**% Bias**	**%reduct |bias|**	**t**	**p>|t|**
**Age**	Before	60.84	56.35	48.50		19.20	0.00
	After	60.73	60.90	-1.90	96.20	-0.85	0.40
**Gender**	Before	0.53	0.48	9.20		3.67	0.00
	After	0.53	0.51	3.10	66.10	1.50	0.13
**Han population**	Before	0.08	0.06	8.50		3.33	0.00
	After	0.07	0.07	0.00	100.00	0.00	1.00
**Married**	Before	0.88	0.91	-11.40		-4.44	0.00
	After	0.88	0.87	3.10	73.20	1.35	0.18
**Education level**	Before	1.01	1.20	-16.60		-6.63	0.00
	After	1.01	0.93	7.40	55.50	3.64	0.00
**Occupation**	Before	1.38	1.24	13.70		5.34	0.00
	After	1.37	1.29	7.80	43.60	3.62	0.00
**Urban/rural location**	Before	0.22	0.18	11.10		4.35	0.00
	After	0.22	0.18	8.30	24.50	3.99	0.00
**Health insurance type**	Before	1.13	1.10	8.10		3.21	0.00
	After	1.13	1.10	7.10	12.80	3.38	0.00
**Household expenditure**	Before	2.04	2.07	-3.40		-1.36	0.17
	After	2.04	2.00	5.00	-47.50	2.41	0.02
**Self-reported health status**	Before	0.57	1.09	-68.00		-27.67	0.00
	After	0.57	0.56	1.70	97.50	0.86	0.39
**Regional location**	Before	0.99	0.85	16.70		6.69	0.00
	After	0.99	0.97	1.80	89.20	0.87	0.39

The order "pstest" in STATA was used to compare individual characteristics between health-shocks respondents and the non-health-shocks respondents before and after propensity score matching.
The first line of each variable showed before matching the distribution, the bias and t-test results of the difference between two groups, which indicated that all the variables except household expenditure were statistically different.
The second line of each variable showed after matching the distribution, the bias and t-test results of the difference between two groups. After matching, all the bias of 11 matching variables were less than 10 (one of the standards of well-balanced), however the t-test results indicated that 5 variables (education level, occupation, urban/rural location, health insurance type and household expenditure) were still statistically different.

Appendix C Individual Characteristics of the Study Sample from CHARLS before and after 1:3 Nearest Matching

		Mean				t-test	
**Variable**		**HS**	**non-HS**	**% Bias**	**%reduct |bias|**	**t**	**p>|t|**
**Age**	Before	60.84	56.35	48.50		19.20	0.00
	After	60.73	60.95	-2.40	95.00	-1.11	0.27
**Gender**	Before	0.53	0.48	9.20		3.67	0.00
	After	0.53	0.51	3.70	59.50	1.79	0.07
**Han population**	Before	0.08	0.06	8.50		3.33	0.00
	After	0.07	0.08	-1.00	88.50	-0.44	0.66
**Married**	Before	0.88	0.91	-11.40		-4.44	0.00
	After	0.88	0.87	2.80	75.80	1.23	0.22
**Education level**	Before	1.01	1.20	-16.60		-6.63	0.00
	After	1.01	0.97	3.50	78.90	1.70	0.04
**Occupation**	Before	1.38	1.24	13.70		5.34	0.00
	After	1.37	1.33	4.50	67.40	2.06	0.04
**Urban/rural location**	Before	0.22	0.18	11.10		4.35	0.00
	After	0.22	0.19	7.30	33.60	3.50	0.00
**Health insurance type**	Before	1.13	1.10	8.10		3.21	0.00
	After	1.13	1.11	6.20	24.30	2.94	0.00
**Household expenditure**	Before	2.04	2.07	-3.40		-1.36	0.17
	After	2.04	2.01	3.60	-6.70	1.75	0.08
**Self-reported health status**	Before	0.57	1.09	-68.00		-27.67	0.00
	After	0.57	0.57	0.90	98.70	0.44	0.66
**Regional location**	Before	0.99	0.85	16.70		6.69	0.00
	After	0.99	0.97	1.60	90.20	0.78	0.43

The order "pstest" in STATA was used to compare individual characteristics between health-shocks respondents and the non-health-shocks respondents before and after propensity score matching.
The first line of each variable showed before matching the distribution, the bias and t-test results of the difference between two groups, which indicated that all the variables except household expenditure were statistically different.
The second line of each variable showed after matching the distribution, the bias and t-test results of the difference between two groups. After matching, all the bias of 11 matching variables were less than 10 (one of the standards of well-balanced), however the t-test results indicated that 4 variables (education level, occupation, urban/rural location and health insurance type) were still statistically different.

Appendix D Individual Characteristics of the Study Sample from CHARLS before and after Radius Matching

		Mean				t-test	
**Variable**		**HS**	**non-HS**	**% Bias**	**%reduct |bias|**	**t**	**p>|t|**
**Age**	Before	60.84	56.35	48.50		19.20	0.00
	After	60.84	56.35	48.50	0.00	8.15	0.00
**Gender**	Before	0.53	0.48	9.20		3.67	0.00
	After	0.53	0.48	9.20	0.00	1.57	0.12
**Han population**	Before	0.08	0.06	8.50		3.33	0.00
	After	0.08	0.06	8.50	0.00	1.37	0.17
**Married**	Before	0.88	0.91	-11.40		-4.44	0.00
	After	0.88	0.91	-11.40	0.00	-1.83	0.04
**Education level**	Before	1.01	1.20	-16.60		-6.63	0.00
	After	1.01	1.20	-16.60	0.00	-2.85	0.00
**Occupation**	Before	1.38	1.24	13.70		5.34	0.00
	After	1.38	1.24	13.70	0.00	2.19	0.03
**Urban/rural location**	Before	0.22	0.18	11.10		4.35	0.00
	After	0.22	0.18	11.10	0.00	1.82	0.02
**Health insurance type**	Before	1.13	1.10	8.10		3.21	0.00
	After	1.13	1.10	8.10	0.00	1.35	0.18
**Household expenditure**	Before	2.04	2.07	-3.40		-1.36	0.17
	After	2.04	2.07	-3.40	0.00	-0.58	0.56
**Self-reported health status**	Before	0.57	1.09	-68.00		-27.67	0.00
	After	0.57	1.09	-68.00	0.00	-12.33	0.00
**Regional location**	Before	0.99	0.85	16.70		6.69	0.00
	After	0.99	0.85	16.70	0.00	2.88	0.00

The order "pstest" in STATA was used to compare individual characteristics between health-shocks respondents and the non-health-shocks respondents before and after propensity score matching.

The first line of each variable showed before matching the distribution, the bias and t-test results of the difference between two groups, which indicated that all the variables except household expenditure were statistically different.

The second line of each variable showed after matching the distribution, the bias and t-test results of the difference between two groups. After matching, only the bias of 5 matching variables were less than 10 (one of the standards of well-balanced), and the t-test results indicated that 7 variables (age, married, education level, occupation, urban/rural location, self-reported health status and regional location) were still statistically different.

Appendix E Sensitivity Analysis: Odds Ratios from Multilevel PSM-DID Regression Models using the 1:3 Nearest Matching

Health Shocks	Smoking	Drinking	Exercise	Physical examination	Basic examination	Auxiliary inspection
**HS×Time**	**0.54****	**0.62*****	**1.18**	**1.12**	**1.11**	**1.18***
HS	0.03***	0.84	1.43***	2.01***	1.83***	2.05***
Time	0.82	1.12	1.49***	1.29***	1.60***	1.45***
**Major HS×Time**	**0.39*****	**0.56*****	**1.20**	**1.10**	**1.05**	**1.14**
Major HS	0.02***	0.64***	1.47***	2.31***	2.04***	2.36***
Time	0.82	1.12	1.49***	1.26***	1.61***	1.42***
**Minor HS×Time**	**0.65****	**0.60*****	**1.34****	**1.18***	1.20	**1.29****
Minor HS	0.04***	0.91	1.28*	1.87***	1.69***	1.87***
Time	0.94	1.16	1.47***	1.27***	1.62***	1.44***
**Cancer×Time**	**0.02****	**0.41***	**0.83**	**1.07**	**1.12**	**1.05**
Cancer	0.06	0.44*	1.76	2.31***	2.13***	2.62***
Time	0.90	1.14	1.50***	1.23***	1.62***	1.40***
**Stroke×Time**	**0.53*****	**0.41*****	**1.29**	**1.15**	**0.97**	**1.32***
Stroke	0.06***	0.39***	1.90***	2.83***	2.46***	2.54***
Time	0.93	1.09	1.46***	1.22***	1.59***	1.40***
**Heart disease×Time**	**0.58****	**0.56*****	**1.27**	**1.09**	**1.15**	**1.08***
Heart Disease	0.35***	0.80	1.25	2.33***	2.06***	2.46***
Time	0.87	1.15	1.45***	1.27**	1.61***	1.43***
**Diabetes×Time**	**0.65**	**0.53*****	**1.35****	**1.28***	**1.39***	**1.36****
Diabetes	0.15***	0.69	1.58**	2.50***	1.84***	2.61***
Time	0.94	1.64	1.46***	1.23***	1.58***	1.40***
**Hypertension×Time**	**0.50****	**0.59*****	**1.41*****	**1.25***	**1.26***	**1.39****
Hypertension	0.04***	0.99	1.16	1.69***	1.67***	1.65***
Time	0.94	1.17	1.49***	1.28***	1.65***	1.45***

The multilevel PSM-DID model was conducted by adjusting age, gender, ethnicity, marital status, education level, occupation, household expenditure, self-reported health status, and regional location, and estimated at individual-wave-level, individual-level, and community-level. HS=health shocks.
**p < .05. **p < .01. ***p < .001.*
